# Evaluation of HNO_3_, SO_2_, and NH_3_ in the Surface Tiled Aerosol and Gaseous Exchange (STAGE) option in the Community Multiscale Air Quality Model version 5.3.2 against field-scale, in situ and satellite observations

**DOI:** 10.5194/gmd-19-9377-2026

**Published:** 2026-10-05

**Authors:** Jesse O. Bash, John T. Walker, Zhiyong Wu, Ian C. Rumsey, Ben Murphy, Christian Hogrefe, Kathleen M. Fahey, Havala O. T. Pye, Matthew R. Jones, K. Wyat Appel, Mark W. Shephard, Najwa I. Alnsour, Karen E. Cady-Periera

**Affiliations:** 1 US Environmental Protection Agency, Research Triangle Park, NC, USA; 2 Center for Ecology and Hydrology, Edinburgh, Scotland, UK; 3 Air Quality Research Division, Environment and Climate Change Canada, Toronto, Ontario, Canada; 4 North Carolina State University, Raleigh, NC, USA; 5 Atmospheric and Environmental Research, Inc., Lexington, MA, USA

## Abstract

The Surface Tiled Aerosol and Gaseous Exchange (STAGE) model was developed as a unified system for estimating dry deposition and bidirectional exchange for field-scale applications and for use within the CMAQ v5.3.2 regional scale model. The field-scale model was evaluated against micrometeorological flux measurements of NH_3_, HNO_3_, and SO_2_ at a managed grassland site at Duke Forest Blackwood Division, NC (35.58° N, 79.05° W) and NH_3_ in a cultivated corn field in Lillington, NC (35.38, 78.78° N). When using data collected at the field-scale for soil and vegetation NH_3_ compensation points, modeled fluxes for all species agreed well with the observations, with mean bias within or near the reported measurement uncertainty. However, when using the default CMAQ v5.3.2 values for NH_3_ emission potentials for soil ($\Gamma_{\text{soil}}=20$) and vegetation ($\Gamma_{\text{apoplast}}=247$) at the Duke Forest grassland site, the model estimated a mean net deposition (−1.3 ng m^−2^ h^−1^) while a mean NH_3_ evasive flux (8.4 ng m^−2^ h^−1^) was observed. An annual 2016 model simulation of CMAQ v5.3.2 was evaluated against Cross-Track Infrared Sounder (CrIS) satellite NH_3_ observations to assess if the box model biases at the field scale where site specific of indicative of more general model biases. The evaluation with CrIS observations shows a broad underestimation of NH_3_ concentrations by approximately 1 to 2 ppb in the U.S. Great Plains. This is in general agreement with the results from the grassland field data indicating that there is likely an underestimation of the evasive NH_3_ flux in grassland sites in CMAQ due to the model’s default tabular values of the vegetation/litter ${\text{NH}}_{4}^{+}$ concentrations. The sensitivities of the STAGE model to the soil and vegetation emission potentials indicates that regional scale model results for NH_3_ can be further improved with additional micrometeorological flux and vegetation and soil chemistry measurements over different land use types, soil types, and vegetation phenological stages.

## 1 Introduction

Ammonia (NH_3_), sulfur dioxide (SO_2_) and nitric acid (HNO_3_) are gaseous precursors to atmospheric particulate matter formation (Seinfeld and Pandis, 1998) which is deleterious to human health and of global importance (Burnett et al., 2018). In addition to adverse human health impacts the atmospheric deposition of these pollutants contributes to soil and water acidification and excess nutrient loading to sensitive ecosystems (Greaver et al., 2012). In the U.S., oxidized forms of nitrogen and sulfur are regulated under the U.S. National Ambient Air Quality Standards (NAAQS) established under the Clean Air Act Amendments (U.S. Congress, 1990; U.S. EPA, 2020). The exchange of atmospheric aerosols and trace gases between the atmosphere and biosphere is an essential process in the source, transport and fate of atmospheric pollutants and represents an important vector of ecosystem and human health exposures (Eschleman and Sabo, 2016; Greaver et al., 2012; Burnett et al., 1998; Galloway et al., 2020). In regional and global chemical transport models, evasive/emission and dry deposition components of the net flux are typically treated separately, often with different parameterizations and assumptions, despite being governed by many of the same biogeochemical and physical processes (Saylor and Hicks, 2016). Field-scale models have been developed that consistently parameterize the net flux of pollutants for a limited number of atmospheric trace gases (Nemitz et al., 2001; Personne et al., 2009; Massad et al., 2010; Stella et al., 2011). In regional and global scale applications, these flux/bidirectional exchange parameterizations have only been applied to mercury (Hg) and NH_3_ (Zhang et al., 2010; Bash 2010; Wichink Kruit et al., 2012; Bash et al., 2013; Wang et al., 2014; Zhu et al., 2015) despite many volatile organic carbon (VOC; e.g., Millet et al., 2018) and nitrogen compounds (e.g. Skiba et al., 1999; Wu et al., 2019) exhibiting both evasive and deposition fluxes depending on environmental and biological conditions.

Models developed to simulate field-scale fluxes often include both evasive and deposition fluxes and typically employ an electrical resistance analog to drive the estimated fluxes in bulk, e.g., big leaf (Nemitz et al., 2001), multilayer Eulerian (e.g. Wolfe and Thornton, 2011, Bash et al., 2010), or Lagrangian frameworks (e.g. Raupauch, 1989). However, regional and global scale chemical transport models typically parameterize deposition and emission processes separately with deposition being typically described using a resistance model analog (e.g. Pleim and Ran, 2011) and emissions typically modeled with a bottom up approach using emissions factors (Olaguer, 2017). These different approaches for modeling emissions and deposition arise due to the differing needs of science and regulatory applications. For example, field-scale models are typically used to illuminate processes governing the exchange of trace pollutants, while regional-scale models like CMAQ are used, among other purposes, to support environmental legislation like the National Ambient Air Quality Standards, NAAQS (Clean Air Act Amendment, U.S. Congress, 1990) and the Total Maximum Daily Load (TMDL) assessments (Clean Water Act, U.S. Congress, 1972) or critical loads (Byrne, 2015). Emissions factors will likely continue to be used to support the accounting of anthropogenic emissions regulated under the NAAQS. However, natural and evaporative emissions where the flux is determined by the production of the trace gas in the environmental media, e.g., soil NO_x_, NH_3_ from agricultural sources, and biogenic VOCs, etc., can be modeled using gradient based methods as advocated by Saylor and Hicks (2016).

In versions of CMAQ prior to v5.3.2, the parametrization for NH_3_ bidirectional exchange added canopy elements to the effective aerodynamic resistance, i.e., adding half the in-canopy resistance to the aerodynamic resistance, to effectively model observations of NH_3_ fluxes over a corn canopy (Pleim et al., 2013). While this simulated fluxes over an agricultural field well, it is not well suited to be applied to species that deposit quickly, e.g., HNO_3_, as it results in smaller dry deposition velocities than the typically observed values largely constrained by the aerodynamic and quasi-laminar boundary layer resistances (Nguyen et al., 2015) and was therefore not applied to other modeled species. This resulted in two parallel resistance models, one for NH_3_ bidirectional exchange and one for all other species implemented in CMAQ. Additionally, observations of deposition velocities exhibit a large range across forested, short vegetation and smooth surfaces (Schrader and Brümmer, 2014; Zhang et al., 2002). The need for land use specific deposition fluxes from regional scale models is being driven by advancements in the identification of vegetation species and plant functional type differences in critical loads responses to nitrogen and sulfur deposition (Clark et al., 2019) and the land use specific transport effectiveness of deposited nitrogen in the watershed to the surface waters where the impact of nutrient enrichments are driving Total Maximum Daily Load (TMDL) assessments (Hood et al., 2021). In a gridded modeling system, a land use specific, or tiled, approach is used as the vastly different nitrogen budget of agricultural ecosystems relative to other land use types can perturb the entire grid cell nitrogen budget when using a bulk parameterization. Here we modify and generalize the commonly used resistance model developed by Nemitz et al. (2001) for field-scale modeling of bidirectional turbulent fluxes (Massad et al., 2010; Personne et al., 2009; Stella et al., 2011; Hansen et al., 2017) for use in modeling field scale trace gas fluxes and in regional scale air-quality applications. This parameterization is available in CMAQ v5.3 and later releases as the Surface Tiled Aerosol and Gaseous Exchange (STAGE) deposition option (Galmarini et al., 2021; Appel et al., 2021). This dry deposition framework is applied to all modeled atmospheric gases in CMAQ, evaluated against observed field-scale NH_3_, SO_2_, and HNO_3_ fluxes, and, on a regional scale, against monitoring networks and satellite NH_3_ observations.

## 2 Methods and Materials

### 2.1 STAGE resistance parameterization

The STAGE deposition option closely follows the widely used Massad et al. (2010) and Nemitz et al. (2001) parameterizations, modified to include the option for a cuticular compensation point and is unique in CMAQ as it employs the same resistance model for bidirectional and unidirectional exchange (Fig. 1).

The flux of a trace gas between the atmosphere and surface is modeled following Nemitz et al. (2001) as:

(1) $$F_{t}=-\frac{C_{a}-C_{z0}}{R_{a}}$$

where, $C_{a}$ is the ambient atmospheric concentration, $C_{z0}$ is the compensation point concentration at the sum of the aerodynamic displacement height (d) and roughness length ($z_{0}$) and $R_{a}$ is the aerodynamic resistance. The compensation point is estimated using a two-layer, soil and canopy model following Nemitz et al. (2001).

(2) $$c_{z0}=\frac{\frac{C_{a}}{R_{a}}+\frac{C_{l}}{R_{b,v}}+\frac{C_{g}}{R_{\text{gnd}}}}{{\left(R_{a}\right)}^{-1}+{\left(R_{b,v}\right)}^{-1}+{\left(R_{\text{gnd}}\right)}^{-1}}$$

$C_{1}$ is the compensation point at the leaf, $C_{g}$ is the soil air-pore space concentration, $R_{b,v}$ is the leaf quasi-laminar boundary layer resistance following Massad et al. (2010), and $R_{\text{gnd}}$ is defined as the sum of the in-canopy resistance, soil quasi-boundary layer resistance and soil resistance ($R_{\text{gnd}}=R_{\text{inc}}+R_{b,s}+R_{\text{soil}}$). $C_{1}$ is estimated by solving for the exchange between the canopy compensation point and the atmosphere, stomata, cuticle and ground following Kirchhoff’s current law (see Nemitz et al., 2000). $C_{1}$ is solved from this system of equations following the work of Nemitz et al. (2001) with the addition of a cuticular compensation point:

(3) $$C_{1}=\frac{\begin{array}{c} \frac{C_{a}}{R_{a}R_{b,v}}+\frac{C_{\text{stom}}}{R_{a}R_{s}+R_{b,v}R_{s}+R_{\text{gnd}}R_{s}}+\\ \frac{C_{\text{cut}}}{R_{a}R_{\text{cut}}+R_{b,v}R_{\text{cut}}+R_{\text{gnd}}R_{\text{cut}}}+\frac{C_{g}}{R_{b,v}R_{\text{gnd}}} \end{array}}{\begin{array}{c} {\left(R_{a}R_{b,v}\right)}^{-1}+{\left(R_{a}R_{s}\right)}^{-1}+{\left(R_{a}R_{\text{cut}}\right)}^{-1}+{\left(R_{b,v}R_{s}\right)}^{-1}+\\ {\left(R_{b,v}R_{\text{cut}}\right)}^{-1}+{\left(R_{b,v}R_{\text{gnd}}\right)}^{-1}+{\left(R_{\text{gnd}}R_{s}\right)}^{-1}+{\left(R_{\text{gnd}}R_{\text{cut}}\right)}^{-1} \end{array}}$$

where, $C_{\text{stom}}$ is the gaseous compensation point concentration in the leaf mesophyll, $C_{\text{cut}}$ is the gaseous compensation point concentration at the cuticular surface, $R_{s}$ is the sum of stomatal and mesophyll resistances, and $R_{\text{cut}}$ is the cuticular resistance. The resistance parameterization algorithms and references are listed in Table 1.

The cuticular and soil pathways can be either dry, wet or snow covered where each pathway is assumed to be parallel.

(4) $$R_{x}=\frac{f_{\text{dry}}}{R_{x,\text{dry}}}+\frac{f_{\text{wet}}}{R_{\text{wet}}}+\frac{f_{\text{snow}}}{R_{\text{snow}}}$$

where $R_{x}$ is either the cuticular or soil resistance, $f_{\text{dry}}$, $f_{\text{wet}}$ and $f_{\text{snow}}$ are the ratio of dry, wet or snow covered surface to the surface area constrained by $f_{\text{dry}}+f_{\text{wet}}+f_{\text{snow}}=1$, $R_{x,\text{dry}}$ is the resistance of the modeled species to dry soil or cuticular surfaces, $R_{\text{wet}}$ is the resistance of the modeled species to deposition to surface bound water droplets, and $R_{\text{snow}}$ is the resistance of the model species to deposition to snow covered surfaces.

If the soil, cuticular and stomatal compensation points are zero, then the transfer coefficient in this resistance model reduces to a typical dry deposition model similar in form to Clifton et al. (2020):

(5) $$V_{d}={\left[R_{a}+\frac{1}{\frac{1}{R_{b,v}+\frac{1}{\frac{1}{R_{\text{cut}}}+\frac{1}{R_{s}}}}+\frac{1}{R_{\text{gnd}}}}\right]}^{-1}$$

where $V_{d}$ is the dry deposition velocity and the flux can be modeled as:

(6) $$F_{t}=-C_{a}V_{d}$$

This allows for the use of this resistance model framework for species that exhibit bidirectional exchange, e.g., NH_3_, and species that do not, e.g., HNO_3_, and has the advantage of having a consistent set of assumptions regarding the deposition and bidirectional exchange of pollutants. Similarly, the evasive portion of the flux in the CMAQ implementation is estimated following Bash et al. (2013) to separate evasive and deposition fluxes.


(7) $$F_{t}=-C_{a}V_{d}+\frac{C_{z0}\;(C_{a}=0.0)}{R_{a}}$$

Splitting deposition and emission processes allows for the application of source apportionment tools to species that exhibit bidirectional exchange and estimate emission sensitivities, e.g., the sensitivity of ambient NH_3_ to agricultural fertilizer applications.

#### 2.1.1 Model generalizations

This work largely follows the modeling work of Massad et al. (2010) and Nemitz et al. (2001) for most of the resistance algorithms describing physical transport processes and for NH_3_ exchange (Table 1). Most of the other chemically dependent resistance parameterizations follow CMAQ M3Dry parameterization (Pleim and Ran, 2011). However, several modifications were made to generalize the resistance model for application to all CMAQ’s modeled species, implementation into a regional scale model, and to harmonize parameterizations and assumptions used in other modules of the CMAQ modeling system. Additionally, a method for determining the cuticular resistance for non-ionic organic trace gases was developed based on the species vapor pressure and is analogous to aerosol partitioning, Sect. S1 in the Supplement, but is not presented in the evaluation due to a lack of observational data. The cuticular resistances of all other trace gases are modeled following Pleim and Ran (2011).

#### 2.1.2 Resistance to wet surfaces

Prior to CMAQ v5.3, the parameterization of deposition to wet canopy surfaces is effectively an instantaneous diffusion into the canopy water. Here we adapted the mechanisms used to model diffusion and adsorption of trace gasses into water droplets in CMAQ’s extendable aqueous-phase chemistry option (AQCHEM-KMT) (Fahey et al., 2017).

(8) $$k_{\text{mt},i}={\left(\frac{r^{2}}{3D_{g,i}}+\frac{4r}{3\sqrt{\frac{8RT}{{\text{MW}}_{i}\pi }}\alpha_{i}}\right)}^{-1}$$

where $D_{g,i}$ is the gaseous diffusivity and $\alpha_{i}$ is the bulk accommodation coefficent for the modeled species, i. Most vascular, non-aquatic plant leaves are hydrophobic and wetted surfaces will be composed of roughly spherical droplets (Barthlott et al., 2016). If we choose the droplet radius, r, as the characteristic length, then this can be converted to a resistance by

(9) $$r_{\text{mt},i}=\left(\frac{r^{2}}{3D_{g,i}}+\frac{4r}{3\sqrt{\frac{8RT}{{\text{MW}}_{i}\pi }}\alpha_{i}}\right)r^{-1}\;\;=\left(\frac{r}{3D_{g,i}}+\frac{4}{3\sqrt{\frac{8RT}{{\text{MW}}_{i}\pi }}\alpha_{i}}\right)$$

Then the overall resistance to cuticular deposition using the two-film theory of Liss and Slater (1974) is:

(10) $$R_{\text{wet},i}=r_{\text{mt},i}+\frac{r_{\text{mt},i}}{H}$$

where H is the dimensionless Henry’s constant. The radius of the water droplet, r, is taken as a constant of 1.9 × 10^−4^ m. This assumes that the wet leaf surfaces have an RH of 100 % and using the leaf wetness model of van Hove and Adema (1996) and a contact angle of 90°, uniform wetness on the canopy and spherical droplets on the hydrophobic leaf surfaces. This adds a mass accommodation term in addition to solubility into the surface resistance resulting in additional physical limits to the maximum deposition rate of highly soluble compounds.

Burkhardt et al. (2009) derived the moisture depth as a function of RH using observed leaf wetness and Brunauer-Emmett-Teller, BET, theory (Brunauer et al., 1938). They report a maximum depth of 100 μm similar to the observations of van Hove and Adema (1996). These studies give the specific liquid volume per leaf area, $\frac{w_{L}}{\text{LAI}}$.

From Burkhardt et al 2009, the fractional leaf wetness derived empirically from observations is:

(11) $$\text{LW}=3.68\times 10^{-4}e^{7.9\;\text{RH}}$$

where RH is the humidity ranging from 0 to 1. Then the depth of liquid water in meters using the BET theory with empirical coefficients to match the results of van Hove and Adema (1996) becomes:

(12) $$w_{L}=3.13\times 10^{-7}\text{LAI}\;e^{5.767\;\text{RH}}$$

Assuming a contact angle of 90°, uniform wetness on the canopy and spherical droplets on the hydrophobic leaf surfaces the radius of the droplets on the leaves, r, is:

(13) $$r=\frac{6\;w_{L}}{\pi \text{LAI}}$$

#### 2.1.3 In-canopy aerodynamic resistance

Here the in-canopy aerodynamic resistance is estimated by integrating the in-canopy eddy diffusivity from the ground surface to the canopy top, $H_{c}$, using the attenuation coefficient of Yi (2008):

(14) $$R_{\text{inc}}=\int_{0}^{H_{c}}\frac{dz}{K_{t}}$$

(15) $$K_{t}=u_{*}^{2}{\left(\frac{d\bar{U}}{dz}\right)}^{-1}$$

Following Yi (2008) the wind speed in the canopy can be modeled as:

(16) $$\bar{U}(z)=\bar{U}(H_{c})e^{\left[\frac{\text{LAI}}{2}\left(\frac{z}{H_{c}}-1\right)\right]}$$

where z is the height above the soil and LAI is the leaf area index. Combining eqations 14, 15 , 16 and defining the aerodynamic resistance as $R_{a}=\frac{\bar{U}(H_{c})}{u_{*}^{2}}$.


(17) $$R_{\text{inc}}=R_{a}\left(e^{\left[\frac{\text{LAI}}{2}\right]}-1\right)$$

This result can also be derived if the cumulative LAI is chosen as the vertical coordinate assuming a uniform LAI distribution. This is similar to the parameterization in Shuttleworth and Wallace (1985) using the momentum attenuation coefficient from Yi (2008).

### 2.2 NH_3_ bidirectional exchange

Several updates were made to the soil processes in the NH_3_ bidirectional exchange algorithms for the STAGE deposition option implementation in CMAQ v5.3.2. The first was specifying a maximum diffusive length in estimating the resistance to emissions or deposition to soil surfaces. Earlier versions of CMAQ used the 1 cm soil layer depth of the P-X land surface model (Pleim and Xui, 1995). This was changed to 2 cm to be consistent with the measurements used to derive the diffusive model (Kondo et al., 1990) and to fall in the middle of the dry layer thickness range of 1 to 3 cm reported by Swenson and Lawrence (2014).

Releases of CMAQ before v5.3 estimated that 55 % of the soil ammonium was in the soil water solution based on measurements with extractants with variable ionic strengths (Cooter et al., 2010). Here, we employ the non-linear ammonium sorption capacities to estimate the ${\text{NH}}_{4}^{+}$ in the soil water solution and available for evasion (Venterea et al., 2015).

(18) $${\text{NH}}_{4,s}^{+}=\frac{\mu {\text{NH}}_{4,\text{aq}}^{+}}{K+{\text{NH}}_{4,\text{aq}}^{+}}$$

where ${\text{NH}}_{4,s}^{+}$ is the ammonium sorbed to soil particles, ${\text{NH}}_{4,\text{aq}}^{+}$ is the ammonium in the soil water solution, μ is the maximum soil sorption capacity and K is the ${\text{NH}}_{4,\text{aq}}^{+}$ concentration where ${\text{NH}}_{4,s}^{+}=0.5\mu$ (Table 2).

### 2.3 Site descriptions and box model simulations

Field-scale flux data collected from 5 July to 23 November 2012 at a managed, unfertilized 15 ha grass field at the Duke Forest Blackwood Division, NC (35.58° N, 79.05° W; Rumsey and Walker, 2016) from 5 July to 23 November 2012 and at a 200 ha fertilized corn field near Lillington, NC (35.38, 78.78° N; Walker et al., 2013) from 29 May to 29 June 2007 were used to evaluate the STAGE model. Micrometeorological flux measurements of NH_3_ and latent heat available at both sites and HNO_3_ and SO_2_ fluxes are available at the Duke Forest site were used to evaluate modeled fluxes. At Lillington, air concentrations and above-canopy vertical gradients of NH_3_ were measured with a continuous flow “AMANDA” (Ammonia Measurement by ANnular Denuder sampling with online Analysis; Wyers et al., 1993) wet denuder system and fluxes were determined using the modified Bowen ratio method (Meyers et al., 1996). At Duke Forest, air concentrations and above-canopy vertical gradients of HNO_3_, SO_2_ and NH_3_ were measured using the Monitor for AeRosols and GAses in ambient air (MARGA, Metrohm-Applikon, the Netherlands) (Rumsey et al., 2014) and fluxes were determined using the aerodynamic gradient methods (Thomas et al., 2009). At both sites, eddy covariance was used to determine fluxes of latent heat using an open-path infrared gas analyzer (LI-COR 7500, LI-COR, Inc., Lincoln Nebraska). LAI was not measured during the flux campaign at Duke Forest but there were measurements of canopy height and LAI (LAI-2000, LI-COR, Inc., Lincoln, Nebraska) during other periods. This data was used to estimate LAI as a function of canopy height. In the spring and summer, it was assumed LAI = 3.25*H*_c_ and in the fall this relationship was assumed to be LAI = 1.35*H*_c_ due to the drying of the canopy and senescence. Methods for biogeochemical measurements and compensation points for Duke Forest are included in the supplemental material and are described by Walker et al. (2013) for Lillington.

A field-scale box model was developed in the R statistical language that utilizes the same resistance parameterizations and structure as the CMAQ FORTRAN model (see supplemental material). Observed soil and vegetation NH_3_ compensation points were set as the median values reported in Walker et al. (2013) and measured at Duke Forest (see supplemental material) and using CMAQ model parameterized values. Latent heat fluxes were estimated by specifying a water vapor stomatal and soil compensation points assuming saturation at the measured leaf and soil temperatures. Fluxes of HNO_3_ and SO_2_ utilized the same modeling framework with compensation points set to zero for all surface media.

The field-scale model was run for two field campaigns to evaluate its performance in capturing NH_3_ and H_2_O bidirectional exchange at agricultural and grassland sites and to evaluate its performance in capturing HNO_3_ and SO_2_ exchange at a grassland site. These simulations utilized the resistance parameterizations indicated here with the exception of the aerodynamic resistance, which was defined as $R_{a}={\text{Pr}}_{0}\frac{\bar{U}}{u_{*}^{2}}$, where ${\text{Pr}}_{0}$ is the turbulent Prandtle number (assumed to be 0.95). Note, this results in a similar parameteization of $R_{a}$ as Pleim and Ran 2011, Table 1, if $\bar{U}$ is estiamted from $u_{*}$ assuming a log linear wind profile. Latent heat fluxes were estimated by assuming that the leaf and soil H_2_O compensation points are the saturation vapor pressure at the canopy and soil temperatures, respectively. A sensitivity simulation was conducted for both field scale sites to assess the sensitivity of the STAGE parameter uncertainty. This sensitivity simulation consisted of perturbing the CMAQ v5.3.2 default minimum stomatal resistance, cuticular resistance, and ground resistance by ±50 %.

### 2.4 Regional scale application

The model developed by Massad et al. (2010) and Nemitz et al. (2001) with the modifications above is designed to be applied to specific land use types. In the CMAQ v5.3.2 application, this is accomplished by estimating a deposition velocity for each land use type in a grid cell and calculating the grid cell total as an area weighted sum.

(19) $$F_{t,\text{grid}}=\sum_{\text{LU}=1}^{N_{\text{LU}}}f_{\text{LU}}F_{t,\text{LU}}$$

where, $F_{t,\text{grid}}$ is the grid scale flux, LU is the land use index, $N_{\text{LU}}$ is the number of land uses in the grid cell, $f_{\text{LU}}$ is the ratio of the land use area to total grid area, and $F_{t,\text{LU}}$ is the land use specific flux. This scheme makes it necessary to estimate land use specific resistances. This is currently accomplished by applying the land use specific surface roughness length, minimum stomatal resistance, and leaf area index values from the P-X land surface model (Pleim and Xiu, 1995) look up tables in the WRF v4.1.1 model (Skamarock et al., 2019) to recalculate the surface friction velocity and stomatal resistance.

Multiple CMAQ model simulations for the year 2016 were used in this evaluation. Meteorological inputs were from a WRF v4.1.1 simulation using the P-X land surface model, FEST-C model simulations (Ran et al., 2019) were used to provide agricultural data to support the bidirectional exchange model, and emissions were based on the 2016 EMP (Emissions Modeling Platform: https://www.epa.gov/air-emissions-modeling/2014-2016-version-7-air-emissions-modeling-platforms, last access: 15 September 2026, Appel et al., 2021) . Additional details regarding the model simulations and CMAQ v5.3.2 model performance with the STAGE deposition option can be found in Appel et al. (2021). In this study, we explore the ammonia bidirectional exchange results and conduct model sensitivities that incorporate observed fertilization rates and observed values for non-agricultural leaf and soil Γ to evaluate the model sensitivity to these factors.

FEST-C model simulations are known to under-estimate annual fertilization rates (Ran et al., 2019). To explore how this model bias impacts CMAQ v5.3.2 ambient NH_3_ concentrations and deposition estimates, USDA Economic Research Services (ERS) reported annual fertilizer data was used to post-process FEST-C output for corn, cotton, soybean, and wheat. EPIC fertilizer rates were adjusted by multiplying the grid cell fertilization rate by the ratio of the USDA ERS reported rate over the average FEST-C rate for the state and crop. For states that did not have reported values, the mean ratio of the USDA ERS rate over the mean FEST-C rate for states that had data was used for the adjustment (Eq. 20). The mean adjustment factor for nitrogen fixing crops, soybeans in this case, and non-nitrogen fixing crops were used to adjust the fertilization rate of these crops respectively.

(20) $${\text{Fert}}_{\text{adjusted}.\text{crop}}=\max\;\;\left(\frac{{\text{Fert}}_{\text{submitted},\text{crop}}}{\frac{1}{n_{\text{crop}}}\sum {\text{Fert}}_{\text{FEST-}C,\text{crop}}}{\text{Fert}}_{\text{FEST-}C,\text{crop}},{\text{Fert}}_{\max,\text{crop}}\right)$$

where ${\text{Fert}}_{\text{adjusted},\text{crop}}$ is the FEST-C grid cell adjusted fertilization rate, ${\text{Fert}}_{\text{Submitted},i}$ is the USDA mean annual application data for the specified crop, in kg ha^−1^, ${\text{Fert}}_{\text{FEST-}C,\text{crop}}$ is the initial FEST-C grid cell fertilization rate for the state being considered, $n_{\text{crop}}$ is the number of grid cells with fertilization use for the specified crop in the state, and ${\text{Fert}}_{\max,\text{crop}}$ is the maximum fertilization rate estimated from EPIC for the crop, typically about 300 kg ha^−1^ for a non-nitrogen fixing crop. Nitrification and mineralization rates are captured from FEST-C and evasion rates are estimated using the resistance model above to calculate the nitrogen balance for the modeled soil layers.

### 2.5 Model Evaluation

At the field scale, hourly modeled fluxes are compared to micrometeorological observations from a fertilized Zea mays field and an unfertilized managed grass field. Mean and median modeled values are evaluated against observations over the complete measurement period for configurations of the box model using parameterizations from the STAGE model in CMAQ v5.3.2 and using observed values (Table 3). The following model cases were simulated to evaluate the default CMAQ v5.3.2 values for the agricultural Lillington, NC and grassland Duke Forest, NC sites.

- LM – STAGE model simulation of Lillington, NC fluxes using the default CMAQ v5.3.2 tabular inputs.
- LO – STAGE model simulation of Lillington, NC fluxes using observed stomatal, dew, and soil NH_3_ emission potentials and minimum stomatal resistance.
- DFM – STAGE model simulations of the Duke Forest, NC fluxes using the default CMAQ v5.3.2 tabular inputs.
- DFOS – STAGE model simulation of Duke Forest, NC fluxes using observed stomatal, dew, and soil NH_3_ emission potentials and minimum stomatal resistance.
- DFOL – STAGE model simulation of Duke Forest, NC fluxes using observed stomatal, dew, and leaf litter NH_3_ emission potentials and minimum stomatal resistance.

Evaluation of regional scale results of this model against network observations has been published elsewhere (Appel et al., 2021). Here we focus on the evaluation of the regional scale model estimates against Cross-Track Infrared Sounder (CrIS) satellite observations, Shephard et al. (2020), and estimate the agricultural nitrogen budget and its sensitivity to the parameterization of the NH_3_ emission potential.

## 3 Results and Discussion

### 3.1 Box Model Evaluation at Field Sites

The STAGE model captured the observed diel variability and magnitude of the HNO_3_ and SO_2_ fluxes well (Fig. 1 and Table 3). The H_2_O fluxes at Lillington, NC (LO H_2_O) and Duke Forest, NC (DM H_2_O) were overestimated by 27.0 % and 38.6 % when modeled with the available site-specific data, respectively, Table 3. The error in the Lillington, NC H_2_O flux is near the high end of estimated error in eddy covariances fluxes reported by Rannik et al. (2016). The evaluation of modeled H_2_O and NH_3_ fluxes at Duke Forest had higher error than those at the Lillington site (Table 3). This is likely influenced by uncertainty introduced by the lower magnitude of the measured fluxes at the Duke Forest site and the lack of LAI and minimum stomatal resistance measurements which govern the cuticular and stomatal exchange processes respectively. The normalized mean bias of the NH_3_ flux at the Duke Forest site of all the model cases, Table 3, were outside the range of the measurement uncertainty of 31 % reported by Rumsey and Walker (2016). However, the DFOS and DFOL cases using observed emission potentials, Table 2, resulted in a reduction of the model biases of the estimated NH_3_ flux with a substantial increase in the correlation over the DFM case. The DFM case underestimated the observed flux and estimated net deposition over the measurement period. The model was better able to capture the net flux when the observed emission potentials were used in the DFOS and DFOL cases resulting in a higher model correlation. In general, the median modeled NH_3_ fluxes generally underestimated the observed median (Table 3). The diel fluxes indicate that model underestimated nighttime and early morning NH_3_ evasive fluxes and captured midday and afternoon fluxes well (Fig. 2b). Sensitivity simulations identified that NH_3_ emission potentials for the stomata and ground (represented by leaf litter or soil) can largely explain the biases in the modeled fluxes (Table 3). These measured compensation points are larger than the values parameterized in Massad et al. (2010) used in CMAQ v5.3.2, Table 2, and model results using these values at the duke forest site resulted in an averaged −1.3 ng m^−2^ h^−1^ deposition flux while the observed flux was a net emission for the measurement period (Table 3). Model sensitivities at this site show that the modeled fluxes are most sensitive to $\Gamma_{\text{apoplast}}$ and our measurements of $\Gamma_{\text{apoplast}}$ at this site are similar in magnitude to other studies (e.g. Wang and Schjoerring, 2012; Wichink Kruit et al., 2007).

The STAGE model captured the magnitude and variability of the observed values at the fertilized corn canopy measured during the Lillington, NC flux campaign with a normalized mean bias of −23.4 % when using the median value for the soil ${\text{NH}}_{4}^{+}$ and pH, 31 689.7 μM and 6.43, respectively, and a $\Gamma_{\text{apoplast}}$ of 153.5 reported by Walker et al. (2013) and 40.5 % when using the CMAQ v5.3.2 parameterizations (Table 3 and Fig. 3). As noted in Walker et al. (2013), the $\Gamma_{\text{apoplast}}$ measurements at this site are much lower than values inferred from micrometeorological measurements, and this may be due to the extraction technique used and/or the growth stage of the crop during which the apoplast chemistry was measured. The modeled NH_3_ flux at these sites is most sensitive to the soil and vegetation emission potentials and the model estimates bound both the Lillington and Duke Forest, NC measurements if the median or mean of measured soil emission potentials reported in Walker et al. (2013) and measured at Duke Forest are used.

A consistent min morning emission peak was observed at the Lillington, NC site (Walker et al., 2013). The emissions appear to be related to the drying of the canopy in the morning and similar morning emissions have been observed for a grass canopy (Wentworth et al., 2014). A high emission potential was measured on the dew present on the leaves in the morning (Table 2). When the dew compensation point was included in the STAGE model, case LO, there was insufficient ammonium in the dew to explain the emission peak, Fig. S1, in agreement with Walker et al. (2013). At the Lillington site, the canopy did not contain dew according to the leaf wetness measurements after 08:00 EST, Fig. S2, and the modeled evasion from dew occurred before this period while the observed morning evasion occurred primarily between 08:00 and 11:00 EST when the canopy was dry (Fig. 3). The soil between plants and rows at the Lillington site was exposed and had a much higher emission potential than the canopy. We speculate that the mid-morning emission peak could be due to the wetting and drying of soil surfaces rich in ${\text{NH}}_{4}^{+}$ that was not captured by the soil moisture probes. These results are in contrast to the dew drying experiments of Wentworth et al. (2014), where they estimated that the evasion of NH_3_ during morning dew evaporation could account for morning increases in NH_3_ at a high elevation grassland field site at Rock Mountain National Park, CO.

The field scale simulations of the Lillington, NC and Duke Forest, NC observations indicate that the STAGE deposition model captures the observed SO_2_, HNO_3_, and NH_3_ fluxes well when measured soil and canopy parameters are used (Table 3). However, when CMAQ v5.3.2 tabular data are used in the simulation the STAGE model fails to capture the magnitude or even the direction of the observed NH_3_ flux at the Duke Forest site. At the Duke Forest site, the sensitivity simulations perturbing the minimum stomatal resistance, cuticular resistance, and ground resistance were unable to capture the direction of the mean observed NH_3_ flux and generally increased the biases of the modeled SO_2_ flux (Table S1). The modeled HNO_3_ flux is generally insensitive to the canopy resistance parameters as it is primarily governed by the aerodynamic resistance (Table S1). At the Lillington site, the modeled NH_3_ flux is sensitive to both the emission potential and the ground resistance indicating the importance of accurately characterizing soils in fertilized agricultural sites for estimating NH_3_ fluxes (Table S1). This is the first evaluation of the CMAQ bidirectional NH_3_ exchange parameterization against non-agricultural flux observations and indicates that the tabular data in CMAQ v5.3.2 do not reflect the ammonia emission potentials at the Duke Forest, NC field site. The model evaluation at the Lillington, NC agricultural is comparable to previous studies (Pleim et al., 2013). Additional measurements of soil and vegetation, ${\text{NH}}_{4}^{+}$, pH, and NH_3_ emission potentials would be beneficial in improving CMAQ model emissions and deposition estimates in non-agricultural regions which accounts for approximately 75 % of terrestrial surfaces of the 12 km conterminous US model domain.

### 3.2 Regional Scale CMAQ Simulations

Annual ambient NH_3_ concentration estimates using the CMAQ modeling system generally capture the magnitude and spatial patterns observed in Cross Infrared Sounder (CrIS) v1.6 (Shephard et al., 2020; Shephard and Cady-Pereira, 2015) satellite observations (Fig. 4). CrIS v1.6 has a detection limit of approximately 0.5 ppb under typical atmospheric conditions (Shephard et al., 2025). Here we limited analysis to CrIS observations made during cloud free days and regions where the CrIS observations are generally greater than 1 ppb (Fig. 4a). CMAQ v5.3.2 model concentrations were paired in space and time with the CrIS observations. Model estimates exceed CrIS observations in the agricultural regions of California, the Upper Midwest, and Eastern North Carolina and underestimate observations in the Great Plains, the leeward side of the Sierra Nevada’s in the Mojave Desert, Eastern Washington, and the agricultural regions of North Central Mexico and coastal areas of Sinaloa Mexico (Fig. 4). The mean overestimates in these areas range from 0.5 to 0.9 ppb with maximum overestimates from 7.1 to 9.5 ppb, while mean underestimates at these sites range from −0.6 to −0.62 ppb with maximum underestimates from −2.6 to −3.8 ppb. EPIC fertilization estimates used in CMAQ (Cooter et al., 2012; Bash et al., 2013) only cover the conterminous United States, while values in the Canadian and Mexican portions of the domain use look up tables for agriculture following Zhang et al. (2010) which likely contribute to the observed biases between CMAQ and the CrIS retrievals in Mexico. CMAQ underestimates CrIS NH_3_ observations by approximately 1 ppb over a large swath of non-agricultural land areas (Fig. 4). The general CMAQ underestimate of CrIS NH_3_ observations in these areas indicates that the CMAQ tabular emission potentials for non-agricultural areas may be generally underestimated as they were for the Duke Forest, NC site. The biases on the leeward side of the Sierra Nevada Mountains where there is little vegetation could be due to the evaporation of NH_4_NO_3_ aerosols which CMAQ underestimates from the intensive agricultural and urban areas upwind (Kelly et al., 2018) as airmasses undergo adiabatic heating. Currently, we lack ambient NH_3_ measurements and measurements of soil and vegetation emission potentials in the Mojave Desert or any other desert regions and can only speculate as to the cause of the model underestimation.

Annual regional scale CMAQ model NH_3_ values evaluated against CrIS observations show biases that are directionally consistent with the box model simulations over both grasslands and agricultural areas indicting that the default tabular values are contributing to the observed model biases. Additionally, regional scale model results in the agricultural regions of California and Midwestern U.S. overestimate the CrIS observations (Fig. 3). When compared to CrIS and AMoN sites model biases are likely to be reduced with an increase in the natural NH_3_ emission potentials for grasslands. Approximately a 30 % increase in the vegetation emission potential and factor a of 5 increase in the soil surface emission potential would better match the observations at the Duke Forest, NC grasslands site, Table 2, and likely improve the evaluation against CrIS observations in the Great Plains. While this would represent a relatively large change from the existing values for Γ in CMAQ, this change is also well within the variability of vegetation and soil ᒥ measurements (Walker et al., 2013; Zhang et al., 2010; Wentworth et al., 2014).

Regional scale evaluations of trace gas deposition rates and processes are currently limited by sparse flux measurement observations. Ambient air-quality monitoring networks and satellite observations can provide an indirect evaluation of dry and wet deposition processes but are often missing key observations needed to better constrain model processes, e.g. soil and vegetation NH_3_ emission potentials ᒥ, or particulate matter observations to constrain the impact that aerosol-gas partitioning has on ambient concentrations. Due to the lack of co-located ${\text{NH}}_{4}^{+}$ aerosol observations, our current ability to evaluate NH_3_ using satellite and network data are limited to areas where aerosol partitioning processes are likely negligible. Thus, the regional scale CMAQ v5.3.2 evaluation against network (Appel et al., 2021) and CrIS observations best constrain areas that exhibit high NH_3_ emissions resulting in high ambient NH_3_ concentrations that are less likely to be impacted by modeled errors in ${\text{NH}}_{4}^{+}$. Additionally, the estimated fluxes can be qualitatively evaluated by examining the nitrogen deposition and emission budget against previously published values. Here we tabulate evasion estimates and compare them to fertilizer estimates using the EPIC model (Ran et al., 2019). For the 2016 simulations of Appel et al. (2021), 10.3 MT of N were estimated to be applied as fertilizer to row crops, of that the bidirectional exchange model in CMAQ estimated that 0.5 MT of N, or 4.8 % annual emission factor, if estimated to be emitted as NH_3_ (2017 NEI). An annual NH_3_ emission factor for mineral fertilizers of 4.8 % is within the range of reported values of Klimont and Brink (2004). Deposition values peaked downwind of agricultural sources at approximately 30 kg N ha^−1^ in Eastern North Carolina (Fig. 5), and are well within measured annual deposition values downwind of agricultural sources (Shen et al., 2016) but higher than the net deposition flux of 10 to 16 kg N ha^−1^ reported by Walker et al. (2014) and Walker et al. (2008) respectively for this region. The CMAQ estimated net NH_3_ flux is deposition in this area yet CMAQ NH_3_ concentration estimates are approximately two to three time higher than CrIS observations indicating that NH_3_ emissions from sources other than bidirectional exchange may be overestimated or CrIS observations are underestimated in Eastern North Carolina.

## 4 Conclusions

A unified dry deposition and bidirectional exchange model was developed for field-scale and CMAQ regional scale models. The STAGE parameterization captured the magnitude and diel variability of observations on the field-scale well when detailed vegetation dynamics and chemistry were available, much like similarly structured field-scale models (Nemitz et al., 2000; Personne et al., 2009; Massad et al., 2010). When implemented in the regional scale CMAQ modeling system, the difference between STAGE and M3Dry (Pleim et al., 2019) deposition models resulted in minor differences in ambient concentrations of most modeled species with the exception of NH_3_ (Appel et al., 2021). This difference is due to the structure of the STAGE model being more similar to that of the non-bidirectional species in the parameterization of Pleim and Ran (2011) than the M3Dry parameterization of NH_3_ bidirectional exchange (Pleim et al., 2013).

Parameterization of vegetation and soil ammonium status and its impact on the NH_3_ emission potential, ᒥ, in CMAQ with the STAGE option appears to be the driver of modeled ambient NH_3_ biases. On the field-scale, the sensitivity in the modeled NH_3_ to soil and vegetation compensation points fall within the variability of the soil and vegetation measurements. On a regional scale, modeled biases are sensitive to the parameterization of soil NH_4_ sorption curve and vegetation compensation point. Values of soil and vegetation Γ at the Duke forest site and in recent field-scale measurements (Zhang et al., 2010) indicate that the parameterization used in the STAGE deposition option in CMAQ based on the Massad et al. (2010) annual N deposition fields for non-agricultural land is at the low end of observed vegetation and soil Γ and is likely underestimating the variability and magnitude of soil and vegetation compensation points, particularly for grasslands (Mattsson et al., 2009; Zhang et al., 2010; Wentworth et al., 2014). The evaluation against CrIS NH_3_ observations shows that the magnitude and spatial patterns in the concentration fields are captured well in CMAQ simulations but ambient concentrations are overestimated in some predominantly agricultural areas. The regional scale model underestimates ambient NH_3_ in non-agricultural largely vegetated areas when compared to CrIS observations (Fig. 3). This is in general agreement with the differences in the soil sorption parameters in the Venterea et al. (2015) model and leaf and vegetation NH_3_ emissions potential parameters measured at Lillington and Duke Forest, NC and the tabular values for soil and vegetation emission potentials in CMAQ (Tables 2 and 3). The magnitude of the CMAQ modeled NH_3_ bias against CrIS observations using the STAGE surface exchange option, typically less than 1 μg m^−3^, can largely be explained by the differences in the tabular values used in CMAQ and the observed values reported here and in other studies (e.g. Zhang et al., 2010) and indicates that nitrogen cycling in the natural environment implicated in the Duke Forest flux measurements (Rumsey et al., 2016) is greater than in the current CMAQ v5.3.2 parameterization. The general sensitivity of the STAGE model to the soil and vegetation emission potentials indicates that regional scale model results for NH_3_ can be further improved with additional micrometeorological flux measurements and additional measurements of the vegetation and soil chemistry over different land use types, soil types, and vegetation phenological stages.

## Supplementary Material

Supplement1

*Supplement.* The supplement related to this article is available online at https://doi.org/10.5194/gmd-19-9377-2026-supplement.

## Figures and Tables

**Figure 1.**
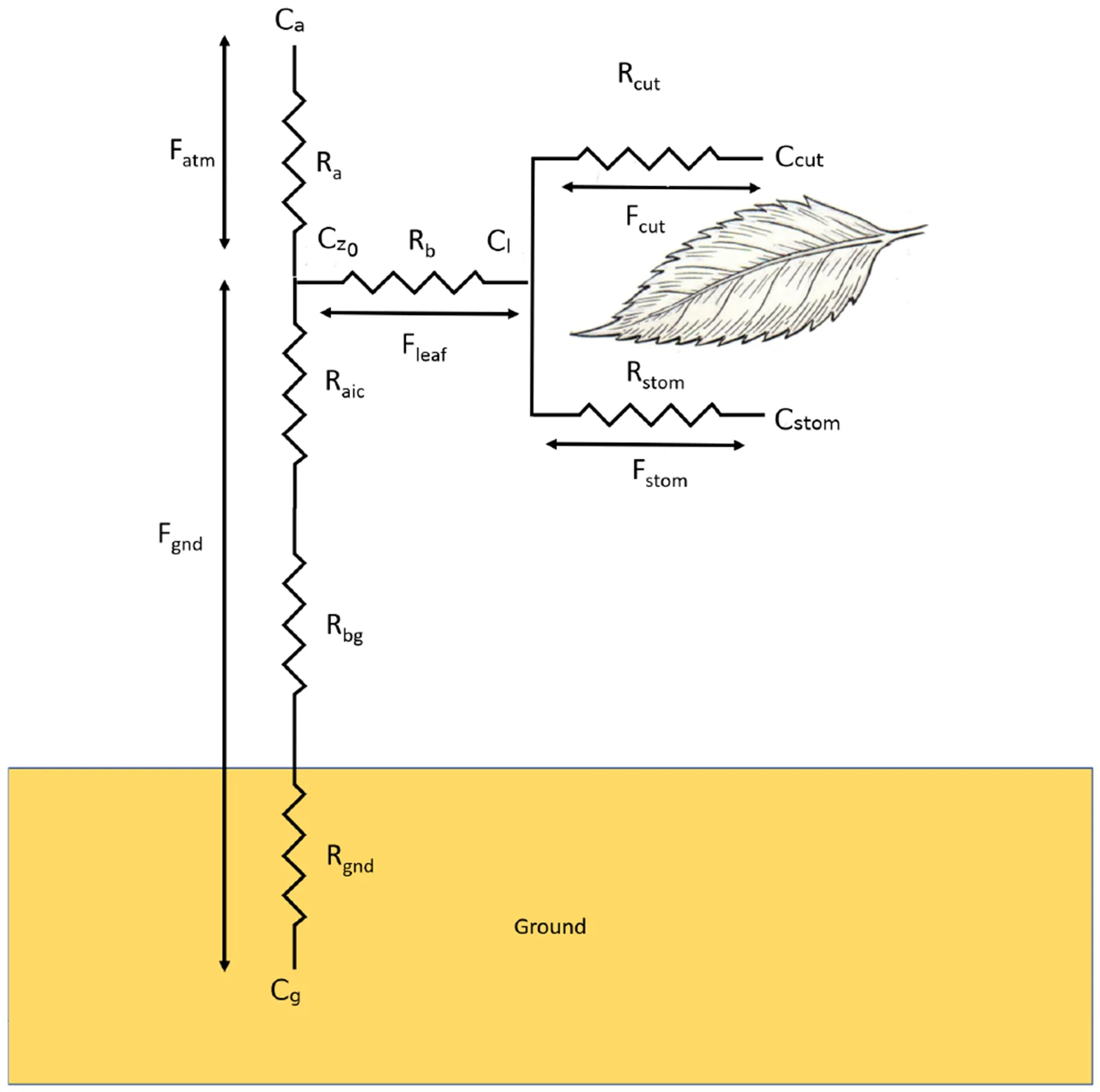
STAGE resistance wire diagram modified from Nemitz et al. (2001) to include non-zero compensation points for leaf cuticular surfaces. Leaf image adapted from Pearson Scott Foresman/Public domain https://commons.wikimedia.org/wiki/File:Acuminate_Leaf_(PSF).jpg (last access: 15 September 2026).

**Figure 2.**
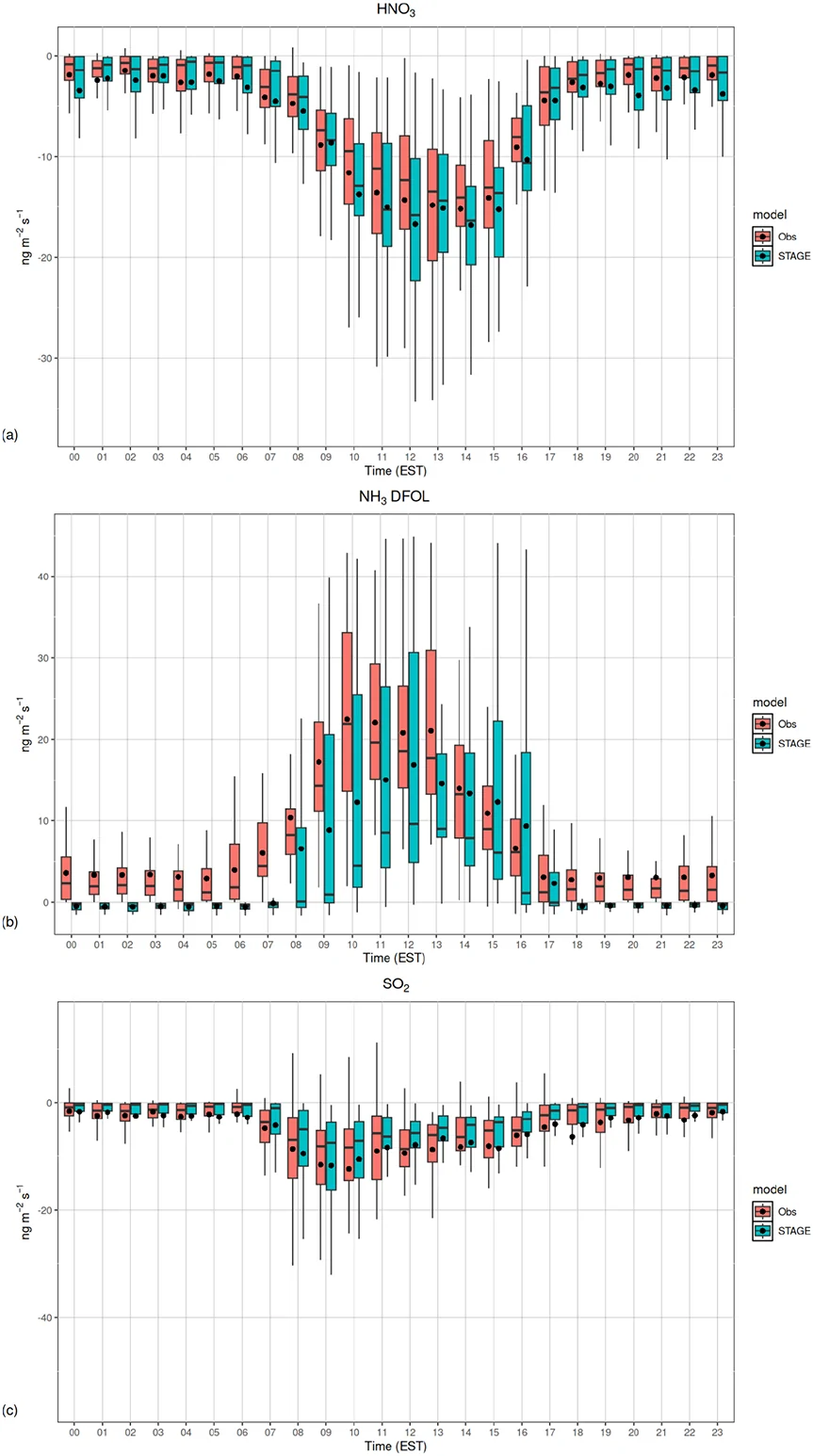
STAGE model estimates (blue) and observations (red) of the HNO_3_ **(a)**, NH_3_ **(b)**, and SO_2_ **(c)** flux measurements taken at the Duke Forest grasslands flux tower. The box bounds the 25th to 75th percentiles, the lines extend to the 5th and 95th percentile, the black horizontal line is the median and the black point represents the mean of the modeled or observed hourly values. Note that negative values indicate net deposition and positive values indicate net evasion.

**Figure 3.**
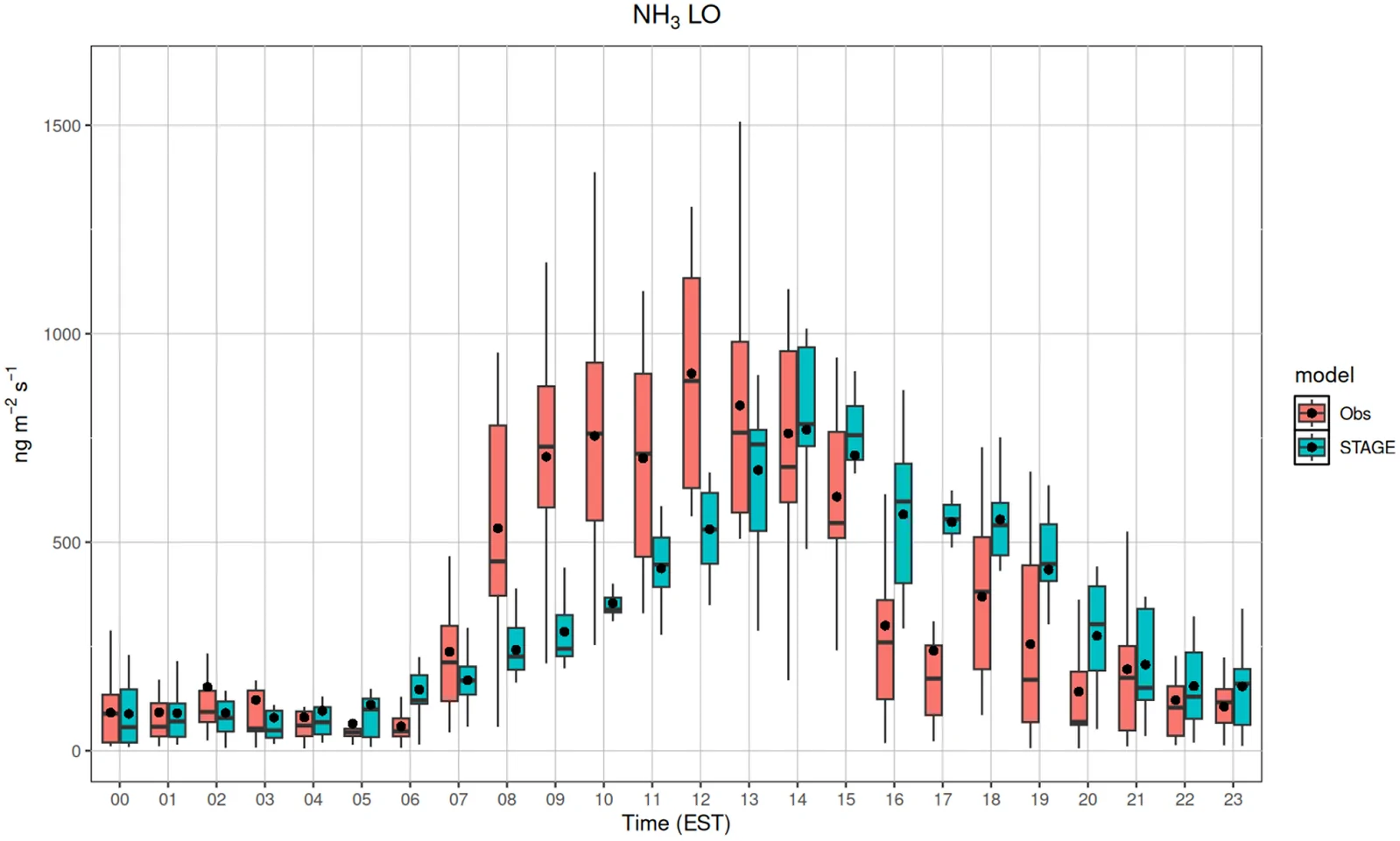
STAGE model estimates (blue) and observations (red) of NH_3_ flux measurements taken at the Lillington, NC flux tower over a fertilized corn canopy. The box bounds the 25th to 75th percentiles, the lines extend to the 5th and 95th percentile, the black horizontal line is the median and the black point represents the mean of the modeled or observed hourly values.

**Figure 4.**
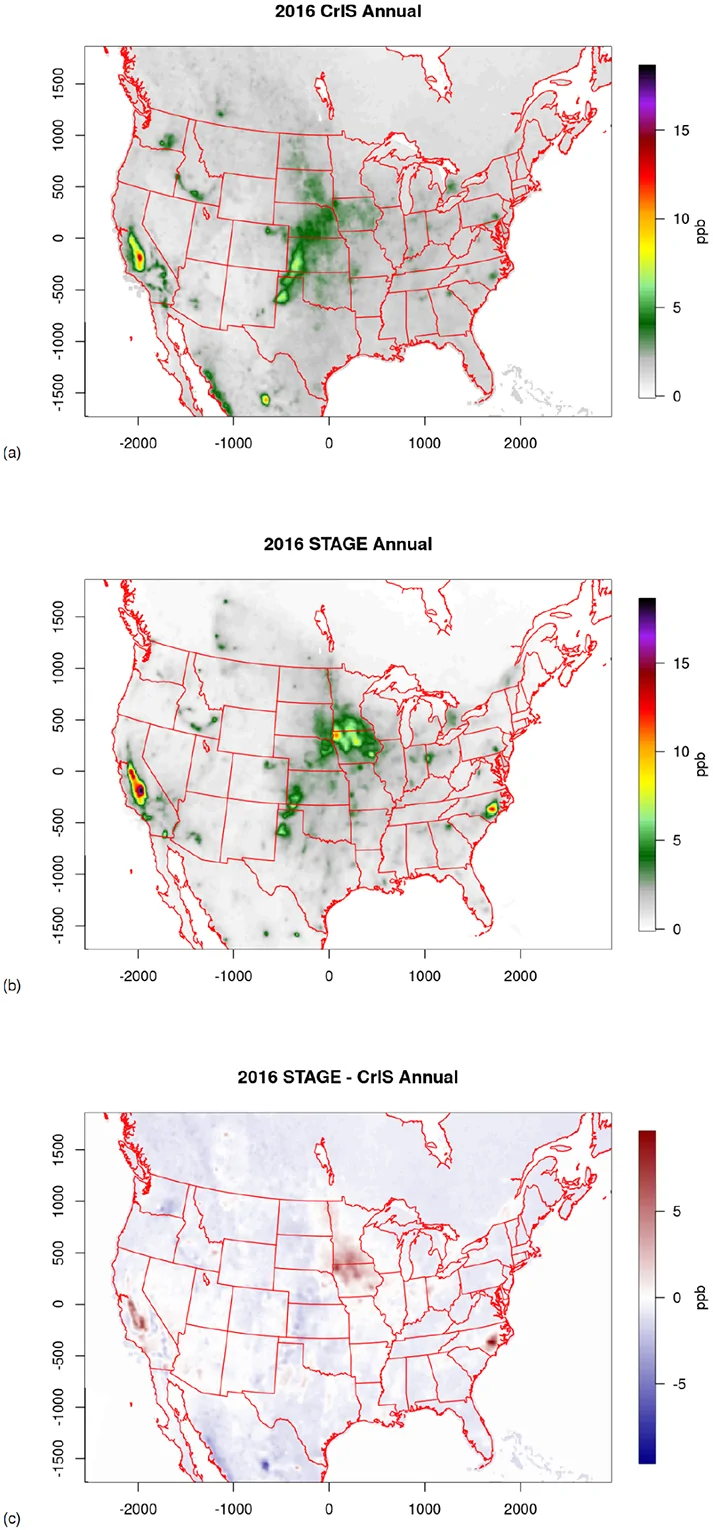
Annual mean Cross Infrared Sounder (CrIS) satellite surface NH_3_ observations binned to CMAQ grid cells for 2016 (Top), mean CMAQ surface NH_3_ estimates paired in space and time with CrIS observations for 2016 (Middle), the mean difference between CMAQ estimates and CrIS observations of NH_3_ paired in space and time for 2016 (Bottom).

**Figure 5.**
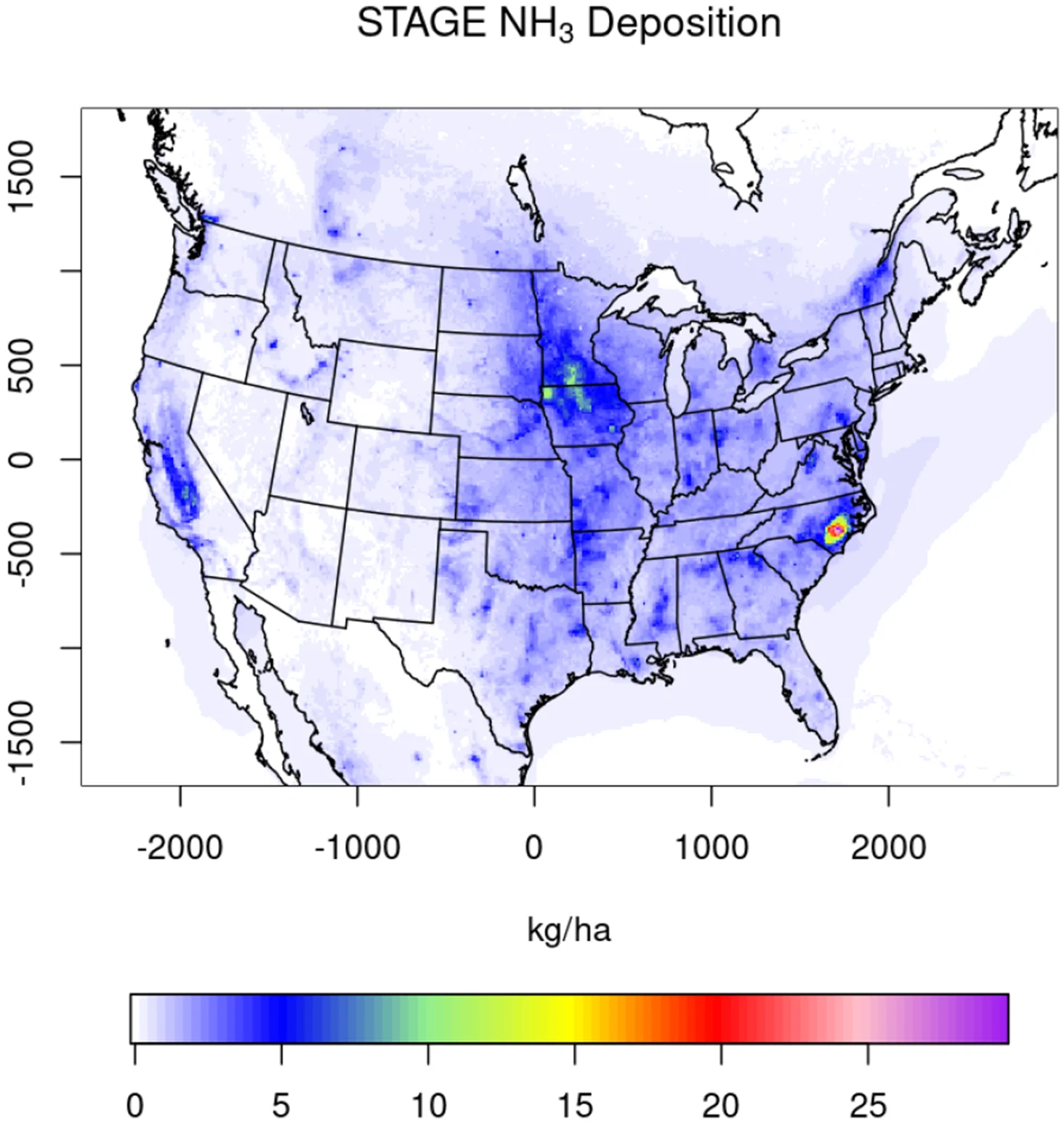
Annual NH_3_ dry deposition, in kg N ha^−1^, for the CMAQ STAGE model simulation evaluated in Appel et al. (2021).

**Table 1.** Modeled resistance the form of the resistance functions and references used in the STAGE model.

Resistance	Formulation	Reference
$r_{a}$	$\frac{\varphi_{h}}{u_{*}k}\left[\text{ln}\left(\frac{z}{z_{0}}\right)-\psi_{h}\left(\frac{z}{L},\frac{z_{0}}{L}\right)\right]$	Pleim and Ran (2011)
$r_{b}$	$\frac{\nu }{D_{i}}{\left(\frac{{\text{clu}}_{*}}{{\text{LAI}}^{2}\nu }\right)}^{\frac{1}{3}}\frac{1}{u_{*}}$	Jensen and Hummelshøj (1995), Massad et al. (2010)
$r_{\text{bg}}$	$\frac{\frac{\nu }{D_{g,i}}-\text{ln}\left({\frac{\delta_{0}}{z}}_{1}\right)}{ku_{*,g}}$	Massad et al. (2010), Nemitz et al. (2001)
$r_{\text{cut},\text{dry}}$	$R_{\text{cut},\text{dry}}=\frac{a_{0}r_{\text{cut}0}}{Rx_{i}}$	Pleim and Ran (2011)
$r_{\text{surf},\text{wet}}$	$R_{\text{surf},\text{wet}}=\left(\frac{l_{\text{wat}}}{D_{g,i}}+\frac{4}{3\sqrt{\frac{4RT}{MW_{i}\pi }}\alpha_{i}}\right)\left(1+\frac{1}{l_{\text{wat}}H_{i}}\right)$	This work, adapted from Fahey et al. (2017)
$r_{\text{stom},i}$	$R_{\text{stom},i}=\frac{D_{g,\text{wat}}}{D_{g,i}}R_{\text{stom},\text{wat}}$	Pleim and Ran (2011)
$r_{\text{mes},i}$	$R_{\text{mes},i}=\frac{1}{\text{LAI}\left(\frac{H_{i}}{3000}+100\;f_{i}\right)}$	Wesely (1989), Pleim and Ran (2011)
$r_{\text{aic}}$	$R_{\text{aic}}=R_{a}\left(e^{\frac{\text{LAI}}{2}}-1\right)$	This work following Raupach (1989)
$r_{\text{gnd}}$	$R_{\text{gnd},\text{dry}}=\frac{a_{0}R_{\text{gnd}0}}{Rx_{i}}$	Pleim and Ran (2011)
$r_{\text{snow}}$	$R_{\text{snow}}=\frac{a_{0}R_{\text{snow}0}}{Rx_{i}}$	Pleim and Ran (2011)
$r_{\text{snow},\text{diff}}$	$R_{\text{snow},\text{diff}}=10$	Pleim and Ran (2011)

**Table 2.** Tabular CMAQ v5.3 model parameters and measured median values with the standard deviation in brackets. The *μ* and *K* values for Duke Forest, NC are from Alnsour (2020). “NA” indicates that the model did not consider litter emission potentials or the lack of leaf litter at the soil surface in the observations.

Measurement period (units)	CMAQ v5.3.2 Grassland	Duke Forest, NC	CMAQ v5.3.2 Agriculture	Lillington, NC
Soil Γ (dimensionless)	20	869 (436)	85 294	85 294 (446 089)
Litter Γ (dimensionless)	NA	144 (236)	NA	NA
Vegetation Γ (dimensionless)	247	2741 (7097)	Function of soil NH_4_ (2750)	153.5 (130)
Dew Γ (dimensionless)	0	1	0	4565 (3059)
Minimum Stomatal resistance (s m^−1^)	100	Not estimated used CMAQ value	70	154
Venterea *μ* (mg kg^−1^)	550	426 (KCl) 585 (H_2_O)	550	Not measured
venterea *K* (mg L^−1^)	345	124 (KCl) 327 (H_2_O)	345	Not measured

**Table 3.** Evaluation of the STAGE model flux estimates compared against Lillington 2007 and Duke Forest 2012 observations. STAGE NH_3_ flux estimates are shown with and without a dew water Γ parameterization.

Measurement period	Species	Model Case (Source of Parameters)	Mean (Median) Observation	Mean (Median) Model	Normalized Mean (Meidan) Bias	Normalized Mean (Median) Error	Pearson’s *r* (Spearman’s *ρ*)
Lillington 2007	H_2_O	LM (CMAQ v5.3.2)	69.1 (21.1) W m^−2^	141.2 (28.7) W m^−2^	104.4 (53.8) %	106.8 (61.7)%	0.928 (0.933)
Lillington (2007)	H_2_O	$R_{\text{st},\text{min}}^{*}$	–	87.8 (21.7) W m^−2^	27.0 (24.7) %	36.6 (40.4) %	0.940 (0.936)
Lillington (2007)	NH_3_	LM (CMAQ v5.3.2)	359 (192) ng m^−2^ h^−1^	503 (390) ng m^−2^ h^−1^	40.5 (25.1)%	76.4 (80.8) %	0.616 (0.737)
Lillington (2007)	NH_3_	LO (Observed stomatal and soil Γ, $R_{\text{st},\text{min}}$)	–	313 (258) ng m^−2^ h^−1^	−12.6 (9.6)%	61.2 (70.9)%	0.515 (0.651)
Duke Forest (2012)	H_2_O	DFM (CMAQ v5.3.2)	29.8 (8.2) W m^−2^	41.2 (7.1) W m^−2^	38.6 (12.8)%	96.5 (118.9)%	0.560 (0.720)
Duke Forest (2012)	SO_2_	DFM (CMAQ v5.3.2)	−5.8 (−2.7) ng m^−2^ h^−1^	−5.6 (−1.8) ng m^−2^ h^−1^	−4.8 (−13.2)%	96.9 (71.0)%	0.411 (0.602)
Duke Forest (2012)	HNO_3_	DFM (CMAQ v5.3.2)	−6.2 (−3.1) ng m^−2^ h^−1^	−7.4 (−3.8) ng m^−2^ h^−1^	19.3 (12.9)%	61.4 (62.0)%	0.705 (0.792)
Duke Forest (2012)	NH_3_	DFM (CMAQ v5.3.2)	8.4 (4.4) ng m^−2^ h^−1^	−1.5 (−0.9) ng m^−2^ h^−1^	−117.6 (−143.5)%	118.0 (143.1)%	−0.007 (−0.277)
Duke Forest (2012)	NH_3_	DFOS (Observed stomatal and soil Γ)	–	8.6 (0.0) ng m^−2^ h^−1^	2.0 (−55.9)%	125.2 (108.8)%	0.437 (0.451)
Duke Forest (2012)	NH_3_	DFOL (Observed stomatal and leaf litter Γ)	–	7.7 (−0.2) ng m^−2^ h^−1^	−8.1 (−67.2) %	127.3 (120.2) %	0.432 (0.313)

* Minimum stomatal resistance ($R_{\text{st},\text{min}}$)

## Data Availability

Hourly meteorological and flux observations at Duke Forest, NC and Lillington NC are available in the Supplement. CMAQ model results are available upon request from KWA. CrIS observations are available upon request from MS.
