# Supplementary material for: Evaluation of HNO3, SO2, and NH3 in the Surface Tiled Aerosol and Gaseous Exchange (STAGE) option in the Community Multiscale Air Quality Model version 5.3.2 against field-scale, in situ and satellite observations
Source: Geosci Model Dev. Author manuscript; Available in PMC 2026 Oct 31. (PMC13720943; doi:10.5194/gmd-19-9377-2026)
Supplement: Supplementary file 1 [file NIHMS2215934-supplement-Supplement1.zip › gmd-19-9377-2026-supplement-title-page.pdf]

Supplement of Geosci. Model Dev., 19, 9377–9393, 2026
https://doi.org/10.5194/gmd-19-9377-2026-supplement
© Author(s) 2026. CC BY 4.0 License.

Geoscientific Model Development
Open Access

*Supplement of*

# Evaluation of $HNO_3$, $SO_2$, and $NH_3$ in the Surface Tiled Aerosol and Gaseous Exchange (STAGE) option in the Community Multiscale Air Quality Model version 5.3.2 against field-scale, in situ and satellite observations

**Jesse O. Bash et al.**

*Correspondence to:* Jesse O. Bash (jesse.o.bash@met.no)

- gmd-19-9377-2026-supplement-title-page.pdf
- egusphere-2025-3536-supplement-version5
  - Bash et al STAGE RevisedSIv5.pdf
  - CMAQ_STAGE_Model_fluxes_HNO3_Duke_Forest.csv
  - CMAQ_STAGE_Model_fluxes_NH3_Duke_Forest.csv
  - CMAQ_STAGE_Model_fluxes_NH3_Lillington.csv
  - CMAQ_STAGE_model_fluxes_SO2_Duke_Forest.csv
  - Meteorological and flux observations at Duke Forest, NC_Final.csv
  - Meteorological and flux observations at Lillington, NC_Final.csv

The copyright of individual parts of the supplement might differ from the article licence.
