# Supplementary material for: Evaluation of HNO3, SO2, and NH3 in the Surface Tiled Aerosol and Gaseous Exchange (STAGE) option in the Community Multiscale Air Quality Model version 5.3.2 against field-scale, in situ and satellite observations
Source: Geosci Model Dev. Author manuscript; Available in PMC 2026 Oct 31. (PMC13720943; doi:10.5194/gmd-19-9377-2026)
Supplement: Supplementary file 1 [file NIHMS2215934-supplement-Supplement1.zip › egusphere-2025-3536-supplement-version5/Bash et al STAGE RevisedSIv5.pdf]

Supplemental Material

S1. Dry cuticular deposition of non-ionic organic species

Deposition of non-ionic organic species to dry leaf cuticles are modeled similar to processes involving the partitioning of semi-volatile gases to organic aerosols. The assumptions of this work are that the transport from the gas phase to the waxy lipids on the leaf surfaces follow Fick's and Raoult's laws. The two-film resistance theory from Liss and Slater, 1974 is used here. Rather than using Henry's law constants to define a partitioning coefficient, Raoult's law is adapted to find an air-cuticular wax equilibrium partitioning coefficient, K_{ca} (mol cuticle/mol air).

$$K_{ca} = \frac{RT}{P_{vap}\widetilde{M}_{wax}} \quad (S1)$$

Where R is the universal gas constant, T is temperature in K, P_{vap} is the vapor pressure of the organic gas, and $\widetilde{M}_{wax}$ is the inverse mole concentration of the wax media the gas is partitioning to in $m^3\ mol^{-1}$. The volatility of organic gas model species in CMAQ is quantified using C^* rather than vapor pressure, so p_{vap} is calculated using:

$$p_{vap} = \frac{RTMW_{org}}{\frac{C^*}{10^9}} \quad (S2)$$

Where MW_{org} is the molecular weight of the organic gas (g mol^{-1}) and C^* is the saturation concentration in $\mu g\ m^{-3}$. For explicit species, the resulting vapor pressure will be equal to that of the corresponding pure component. For lumped model species, the vapor pressure is representative of the mix of compounds described by that species. $\widetilde{M}_{wax}$ is parameterized from the measurements in Schreiber and Shoenherr 2009.

$$\widetilde{M}_{wax} = \frac{C_{wax}}{M_{wax}} \quad (S3)$$

Where C_{wax} is the inverse mass concentration of epicuticular wax available for partitioning ($m^3\ g^{-1}$) and M_{wax} is the molecular weight of the wax. Here we used the mean measured value, 0.72 g m^{-2}, of wax per unit area of leaf and epicuticular wax thickness, $4.33x10^{-6}$ m from Schreiber and Shoenherr 2009. We then assumed that 10% of this was available for partitioning / the characteristic length based on the range of 10% to 80% in Schreiber and Shoenherr 2009. This resulted in C_{wax} of $1.67x10^3\ g\ m^{-3}$. This is similar to the measurements by Jetter and Reiderer 2016. The epicuticular wax was assumed to be represented by nonacosan-10-ol a common epicuticular wax (Jetter and Reiderer 1994) with a molecular weight of 424 g/mol. Thus $\widetilde{M}_{wax}$ is estimated to be $2.55x10^{-2}\ m^3\ mol^{-1}$. This resulted in a nearly linear relationship with C^* where the

leaf level cuticular resistance in s^{-1} m can be estimated for non-oxidative organic vapors as $R_{cut} = 12.7\ C^{*\ 0.933}$ with an R^2 of 0.992.

The two-film resistance theory from Liss and Slater, 1974 state that the air-film flux can be represented by:

$$F = -\frac{1}{r_{atm} + \frac{r_{leaf}}{K_{ca}}}\left(\chi_{atm} - \frac{\chi_{cut}}{K_{ca}}\right) = -\frac{1}{r_{atm}K_{ca} + r_{leaf}}(\chi_{atm}K_{ca} - \chi_{cut}) \qquad (S4)$$

Where r_{atm} is the atmospheric resistance, r_{leaf} is the resistance to bulk uptake in the leaf, χ_{atm} is the ambient atmospheric pollutant concentration, and χ_{cut} is the concentration in the bulk cuticle. In the STAGE code we assume that r_{atm} is negligible because we are modeling the concentration at the leaf surface. r_{leaf} is estimated as the ratio of the depth of the bulk leaf epicuticular material that participates in pollutant uptake and the diffusion of the pollutant through the epicuticular wax. Note that is analogous to Equ. 7 and includes diffusivity and an accommodation coefficient. Thus, in STAGE the flux is represented as:

$$F = -\frac{K_{ca}}{r_{leaf}}\left(\chi_{atm} - \frac{\chi_{cut}}{K_{ca}}\right) \qquad (S5)$$

Note that for most species χ_{cut} is assumed to be 0 (the species is infinitely dilute in the leaf cuticle) and it would require a model or specification of χ_{cut} to make the model bidirectional.

S2. Soil and vegetation biogeochemistry at the Duke Forest Blackwood Division Site

S2.1 Dew chemistry

Dew droplets and guttation were sampled directly from the grass canopy into a 15 mL syringe on two occasions in 2008. During each event, approximately 15 mL of sample was collected in triplicate from various locations in the field. Samples were analyzed directly for pH and soluble ions as described by Walker et al., 2013.

S2.2 Grass and litter gammas

Live grass and leaf litter on the soil surfaces were collected in five 1m x 1m plots within the study area during summer and fall, 2017. Approximately 50g of material was collected during each sampling event from each plot, of which 5g was subsampled, ground in liquid nitrogen using a mortar and pestle and small coffee grinder then extracted with 25mL of deionized water. pH was determined directly on the extracts. [NH_4^+] in the extracts, which reflects the bulk tissue concentration, was determined by colorimetry after separation of the NH_4^+ from the solution as NH_3 using headspace equilibration. For the headspace method, 5mL of tissue extract was added to a 250mL high density polyethylene jar containing two ALPHA passive samplers (Center for

Ecology and Hydrology; Tang et al., 2001), without the diffusion barrier, affixed to the interior of the lid. The jar was sealed and 5mL of 0.3 N NaOH was added to the extract via septum. NH_3 liberated from the liquid extract into the headspace was collected by the passive diffusion samplers over a period of 48 hours, after which the passive sampler was extracted with 10mL of deionized water. NH_4^+ in the extracts was determined by colorimetry using a Salicylate Method – QuickChem®10-107-06-2-A.

Each batch of vegetation analyses included 5 extracted blanks determined by adding 5 mL of deionized water to the headspace jar along with 5 mL of 0.3 N NaOH and otherwise processed as normal vegetation samples. Extracted blank concentrations (NH_4^+) were averaged and subtracted from vegetation sample results. Each batch of vegetation analyses also included a set of extracted standards to evaluate headspace recovery of NH_4^+. Extracted recovery standards consisted of adding 5 mL of a standard containing 5 $\mu g\ mL^{-1}$ NH_4^+ to the headspace jar along with 5 mL of 0.3 N NaOH and were otherwise processed as normal vegetation samples. Recoveries were >90% on average.

Emission potentials of the grass (Γ) was estimated directly from measured concentrations of $[H^+]$ (M) and $[NH_4^+]$ ($\mu g\ g^{-1}$ tissue fresh weight) in the bulk tissue as:

$$\Gamma = \frac{[NH_4^+] \times (5.56E^{-5}) \times LD}{[H^+]} \qquad (S5)$$

where LD is leaf density ($kg\ L^{-1}$ fresh tissue, equivalent to $g\ cm^{-3}$ fresh tissue). For grass species, the median LD (0.156 $kg\ L^{-1}$) of the 16 C3 species reported by (Sugiyama, 2005) is used. Leaf densities for litter assumed the value for live grass. The factor of 5.56E-5 in the equation for Γ is necessary to convert $[NH_4^+]$ from $\mu g\ NH_4^+\ g^{-1}$ tissue to mol NH_4^+ kg $tissue^{-1}$.

S2.3 Soil gamma

Soil samples were collected (0-3cm depth) in the same five 1m x 1m plots as the vegetation samples during summer and fall, 2017. Approximately 50g of soil was collected and composited during each sampling event in each plot. Samples were immediately placed on ice, returned to laboratory and stored under refrigeration until extraction, typically within 24 hours. Soils were sieved to remove material > 2mm immediately prior to extraction. For extraction of NH_4^+, 5g of field moist soil was added to 25 mL of 0.01M $CaCl_2$, rotated for 30 minutes to mix, centrifuged for 5 minutes and approximately 15 mL of supernatant collected and frozen until analysis. As with the vegetation extracts, NH_4^+ was determined by colorimetry using a Salicylate Method – QuickChem®10-107-06-2-A. pH was determined directly in a 1:1 soil/water slurry. Gravimetric moisture was determined by drying 10g of soil at 100 ºC for 24 hours. The bulk soil emission potential (Γ) was estimated as the molar ratio of NH_4^+ to H^+ (Nemitz et al., 2001).

S3 Model Sensitivity Simulations

Table S1, STAGE model sensitivity where the default CMAQ v5.3.2 values for the minimum stomatal (Rst_min), cuticular (Rcut), and ground surface (Rgnd) resistances were reduced by 50% or increased by 50%, the normalized mean bias is shown in percent and the Person's correlation coefficient is in the bracket.

	Lillington, NC NH_3	Duke Forest, NC NH_3	Duke Forest, NC SO_2	Duke Forest, NC HNO_3
CMAQ v5.3.2	40.5% (0.616)	-117.6% (-0.007)	-4.8% (0.411)	19.3% (0.705)
50% Rst_min	49.1% (0.625)	-112.5% (0.116)	7.2% (0.416)	19.4% (0.705)
150% Rst_min	37.0% (0.611)	-118.7% (-0.135)	-8.1% (0.406)	19.3% (0.705)
50% Rcut	36.1% (0.616)	-119.5% (-0.064)	19.4% (0.401)	21.5% (0.705)
150% Rcut	43.3% (0.615)	-116.1% (-0.072)	-14.7% (0.415)	17.3% (0.704)
50% Rgnd	157.0% (0.593)	-127.4% (-0.276)	23.5% (0.401)	24.6% (0.704)
150% Rgnd	0.6% (0.632)	-113.5% (0.040)	-15.0% (0.413)	17.4% (0.705)
LO, DFOS	-12.6% (0.515)	2.0% (0.437)	-4.8% (0.411)	19.3% (0.705)

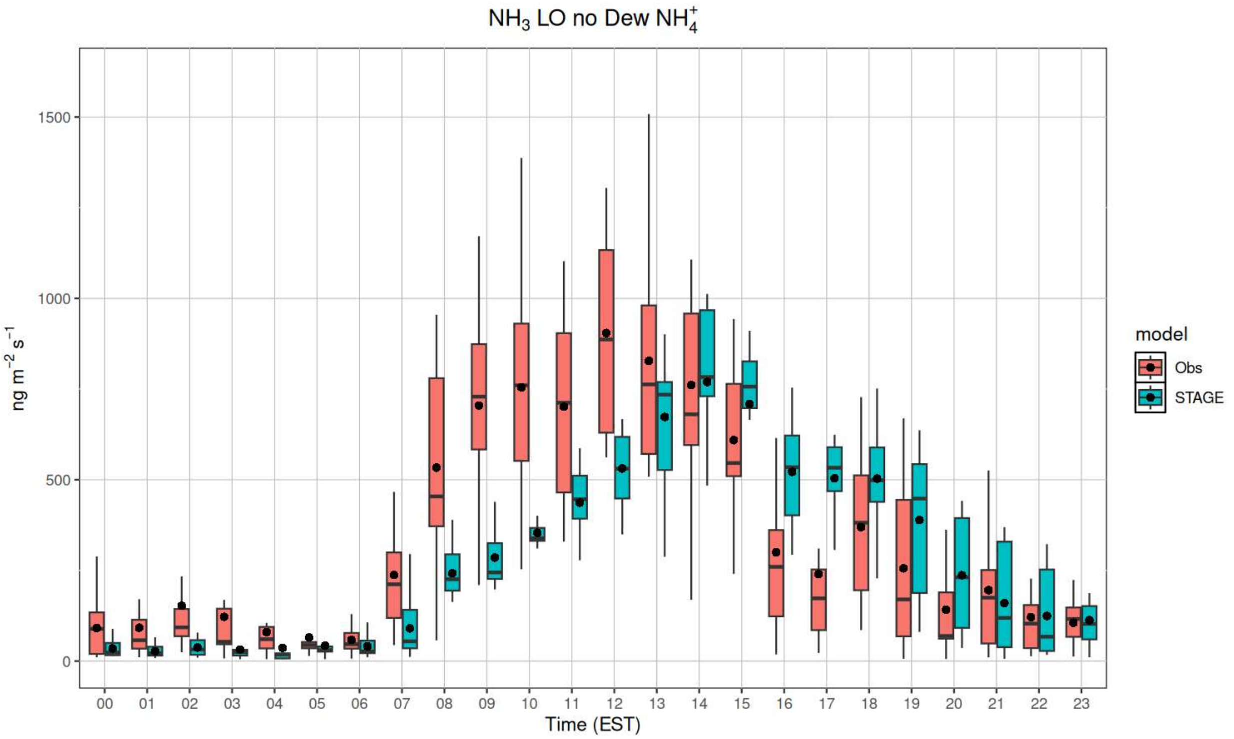

Figure S1, STAGE model estimates (blue) and observations (red) of NH_3 flux measurements taken at the Lillington, NC flux tower over a fertilized corn canopy with the leaf cuticular dew emission potential set to zero (Γ_{dew} =0.0). The box bounds the 25th to 75th percentiles, the lines extend to the 5th and 95th percentile, the black horizontal line is the median and the black point represents the mean of the modeled or observed hourly values.

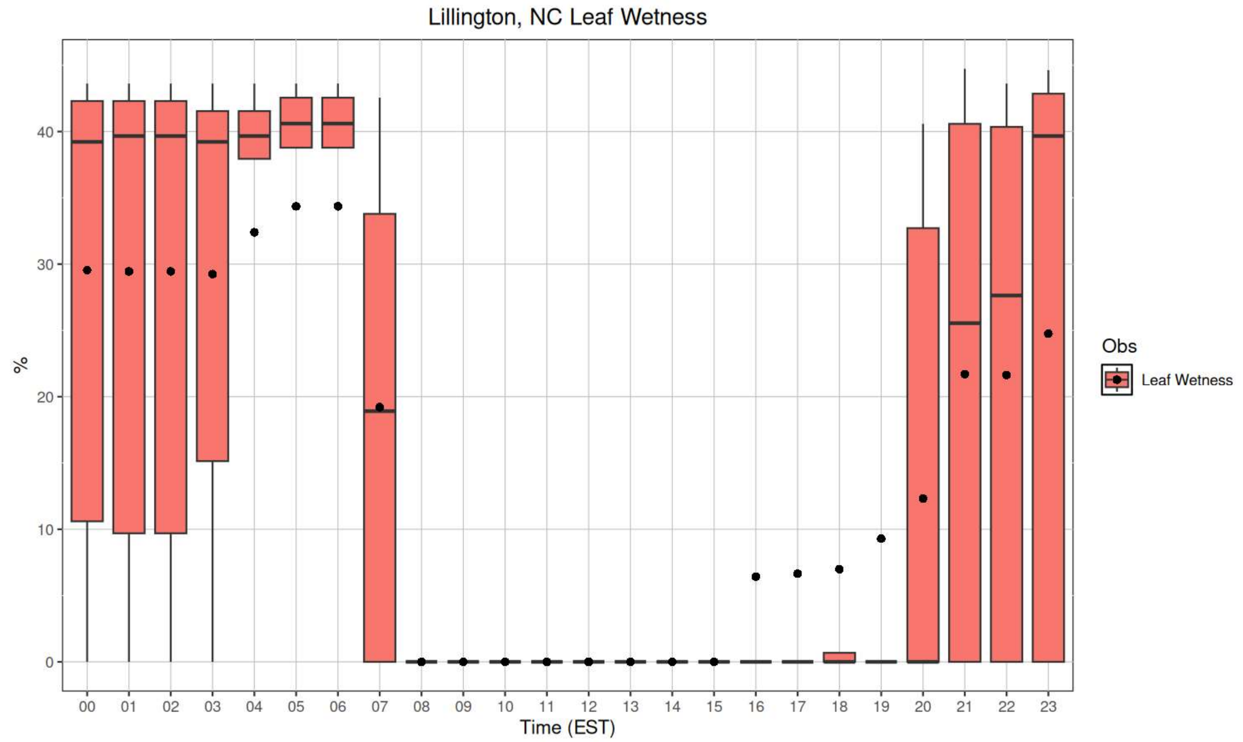

Figure S2, the estimated fraction of the canopy with dew coverage measured by the leaf wetness sensor by hour of the day (local time). The box bounds the 25th to 75th percentiles, the lines extend to the 5th and 95th percentile, the black horizontal line is the median and the black point represents the mean of the modeled or observed hourly values.

References

Jetter, R., Riederer, M., Epicuticular crystals of nonacosan-10-ol: in-vitro reconstitution of factors influencing crystal habits, *Planta*, 195, 257-270, www.jstor.org/stable/23383332, 1994

Jetter, R., Riederer, M., Localization of transpiration barrier in the epi- and intracuticular waxes of eight plant species: water transport resistances are associated with fatty acyl rather than alicyclic components, *Plant Physiology*,170, 921-934, https://doi.org/10.1104/pp.15.01699, 2016

Liss, P.S., Slater, P.G.: Fluxes of gases across the air-sea interface, *Nature*, 247, 181-184, https://doi.org/10.1038/247181a0, 1974

Nemitz, E., Milford, C., Sutton, M.A.: A two-layer canopy compensation point model for describing bi-directional biosphere-atmosphere exchange of ammonia, *Quarterly Journal of the Royal Meteorological Society*, 127, 815–833, 2001

Shreiber, L., Schönherr, J., *Water and solute permeability of plant cuticles. Measurement and data analysis*. Berlin: Springer-Verlag, 2009

Sugiyama, S.: Developmental basis of interspecific differences in leaf size and specific leaf area among C_3 grass species. *Functional Ecology*, 19, 916-924, 2005

Tang, Y.S.; Cape, J.N.; Sutton, M.A.: Development and types of passive samplers for monitoring atmospheric NO_2 and NH_3 concentrations; *The Scientific World*, 1, 513 – 529, 2001

Walker, J.T., Jones, M.R., Bash, J.O., Myles, L., Meyers, T., Schwede, D., Herrick, J., Nemitz, E., Robarge, W.: Processes of ammonia air-surface exchange in a fertilized *Zea mays* canopy. *Biogeosciences*, 10, 981-998, 2013
